# Excitation wavelength optimization improves photostability of ASAP-family GEVIs

**DOI:** 10.1186/s13041-018-0374-7

**Published:** 2018-06-04

**Authors:** Fang Xu, Dong-Qing Shi, Pak-Ming Lau, Michael Z. Lin, Guo-Qiang Bi

**Affiliations:** 1 CAS Key Laboratory of Brain Function and Disease, and School of Life Sciences, University of Science and Technology of China, Hefei, China; 2 Hefei National Laboratory for Physical Sciences at the Microscale, CAS Center for Excellence in Brain Science and Intelligence Technology, and School of Life Sciences, University of Science and Technology of China, Hefei, China; 3 Departments of Neurobiology, Bioengineering and Pediatrics, Stanford University, Stanford, CA 94305 USA

**Keywords:** Genetically encoded voltage indicator, GEVI, Photoswitching, Photostability, ASAP

## Abstract

Recent interest in high-throughput recording of neuronal activity has motivated rapid improvements in genetically encoded calcium or voltage indicators (GECIs or GEVIs) for all-optical electrophysiology. Among these probes, the ASAPs, a series of voltage indicators based on a variant of circularly permuted green fluorescent protein (cpGFP) and a conjugated voltage sensitive domain (VSD), are capable of detecting both action potentials and subthreshold neuronal activities. Here we show that the ASAPs, when excited by blue light, undergo reversible photobleaching. We find that this fluorescence loss induced by excitation with 470-nm light can be substantially reversed by low-intensity 405-nm light. We demonstrate that 405-nm and 470-nm co-illumination significantly improved brightness and thereby signal-to-noise ratios during voltage imaging compared to 470-nm illumination alone. Illumination with a single wavelength of 440-nm light also produced similar improvements. We hypothesize that reversible photobleaching is related to cis-trans isomerization and protonation of the GFP chromophore of ASAP proteins. Amino acids that influence chromophore isomerization are potential targets of point mutations for future improvements.

**Electronic supplementary material:**

The online version of this article (10.1186/s13041-018-0374-7) contains supplementary material, which is available to authorized users.

## Introduction

Understanding neural circuit function requires untangling contextual neural activity, ideally by simultaneous monitoring the activation of all individual neurons in a population. To achieve this goal, various techniques have been developed for large-scale detection of neuronal activity, including multi-electrode recording [1, 2] and optical imaging with calcium indicators [3–7]. Multi-electrode recording provides ultrahigh temporal resolution but lacks cellular specificity, while calcium imaging provides cellular specificity and high spatial accuracy but lacks temporal resolution. Recent years have seen rapid development of genetically-encoded voltage indicators (GEVIs), which enable high spatial and temporal resolution recording of neuronal activities with cell-type specificity [7–9]. Some GEVI mechanisms include fluorescence change upon conformational change of linked voltage-sensing domains (VSDs) [10–13], voltage-sensing opsins [8, 14] and their eFRET-based compounds [15, 16]. As an example of the first mechanism, the recently developed ASAPs are designed as a split voltage-sensing domain (VSD) adapted from voltage-sensitive phosphatase of *Gallus gallus* and a circular-permuted green fluorescent protein (cpGFP) [12, 13]. To follow voltage dynamics in neurons by optical imaging, fast imaging sampling rates and thus short exposure times are required compared to calcium imaging. This necessitates higher excitation powers, which can accelerate indicator photobleaching. Photobleaching reduces apparent brightness of the indicator over the time, impacting the signal-noise ratio (SNR) for detecting superthreshold spiking or subthreshold potential changes. Among GEVIs, ASAPs are unique for their fast kinetics, high response, and compatibility with in vivo two-photon imaging [9, 13, 17]. However, ASAPs, like most GFP-based probes, demonstrate significant fluorescence loss or photobleaching [13] which can be a limitation especially for long-duration imaging experiments. We thus explored the possibility of improving voltage imaging of ASAPs by improving their photostability.

## Materials and methods

### Plasmid construction

All plasmids were constructed following standard molecular biology methods and then verified by sequencing of all cloned fragments. Original pcDNA3.1/Puro-CAG-ASAP1 was obtained from Addgene (Plasmid #52519), and the other variants (ASAP2f, ASAP2f-LE, ASAP2f-V) were cloned between the NheI and HindIII sites.

### HEK 293 cell culture

HEK 293 cells were cultured in Dulbecco’s modified Eagle’s medium (DMEM, Biowhittaker) supplemented with 2 mM L-Glutamine (Gibco), 1 mM Sodium Pyruvate (Gibco) and 10% fetal bovine serum (FBS, TBD Science). Cells were transfected one day after plated onto coverslips (Φ18 mm; Deckglasser) and imaged 2 days after transfection.

### Primary cultures of rat hippocampal neurons

All animal experiments were performed following guidelines and with approval from the Animal Experiments Committee of the University of Science and Technology of China. Primary hippocampal neurons were prepared as previously described [18]. Neurons were transfected with ASAP variants at 10~ 12 days in vitro (DIV) and used 2 days after transfection for imaging and patch-clamp recording.

### Transfection

HEK 293 cells and neurons were transfected with plasmid DNA using a high-efficiency transfection method based on optimized calcium phosphate precipitation [19].

### Imaging

Images of HEK293 cells or neurons were acquired with an inverted microscope (IX71, Olympus) equipped with a 100×/1.45-NA oil-immersion objective. Cells were illuminated with high-power light-emitting diodes with wavelength of 405 nm (M405LP1, Thorlabs), 450 nm (M450LP1, Thorlabs) and/or 470 nm (M470 L3, Thorlabs). 405-nm and 470-nm illumination light were filtered at the exit of LEDs with band-pass filters centered at 405 nm (FBH405–10, Thorlabs) and 475 nm (FF01–475/28, Semrock), respectively. 450 nm illumination was filtered with a band-pass filters centered at 434 nm to allow the 440-nm component passing through. The fluorescence was collected through a 495-nm dichroic mirror (FF495-Di03, Semrock) and a 535/50 emission filter (FF01–535/50, Semrock). Illumination intensity was 5 mW/mm^2^ in all cases for 470-nm light and and 0.1~ 2.5 mW/mm^2^ for 405-nm light at the specimen plane. In all experiments, images were acquired continuously unless described explicitly, at 200 Hz with a sCMOS camera (Zyla, Andor). The camera and the microscope were connected with a 0.35×-magnification adapter (Olympus). Synchronization of electrophysiology and imaging was implemented with a DAQ board (PCI-6229, National Instruments) interfaced with Igor Pro (Wavemetrics) and Micro-Manager [20].

### Electrophysiology

Whole-cell recordings of neurons were carried out using patch-clamp amplifiers (MultiClamp 700B, Molecular Devices) at 25 °C. Neurons were perfused in a chamber mounted on the microscope stage with the extracellular bath solution containing: 150 mM NaCl, 3 mM KCl, 3 mM CaCl_2_, 2 mM MgCl_2_, 10 mM HEPES, and 5 mM Glucose. Borosilicate glass pipettes (4~ 6 MΩ open-tip resistance) were filled with intracellular solution containing: 130 mM K-gluconate, 6 mM NaCl, 20 mM HEPES, 0.2 mM EGTA, 1 mM MgCl_2_, 2 mM MgATP and 0.3 mM Na_3_GTP. Both solution were adjusted to pH 7.3. To induce action potentials, 800~ 1600 pA of current was injected for 1 ms to the neurons. For evaluating the voltage responses to single APs, a data set of ≥6 neurons were obtained with ≥50 APs for each neuron.

### Image analysis

All image analysis was performed with custom ImageJ (NIH) and Matlab (Mathworks) programs.

**Fig. 1:**
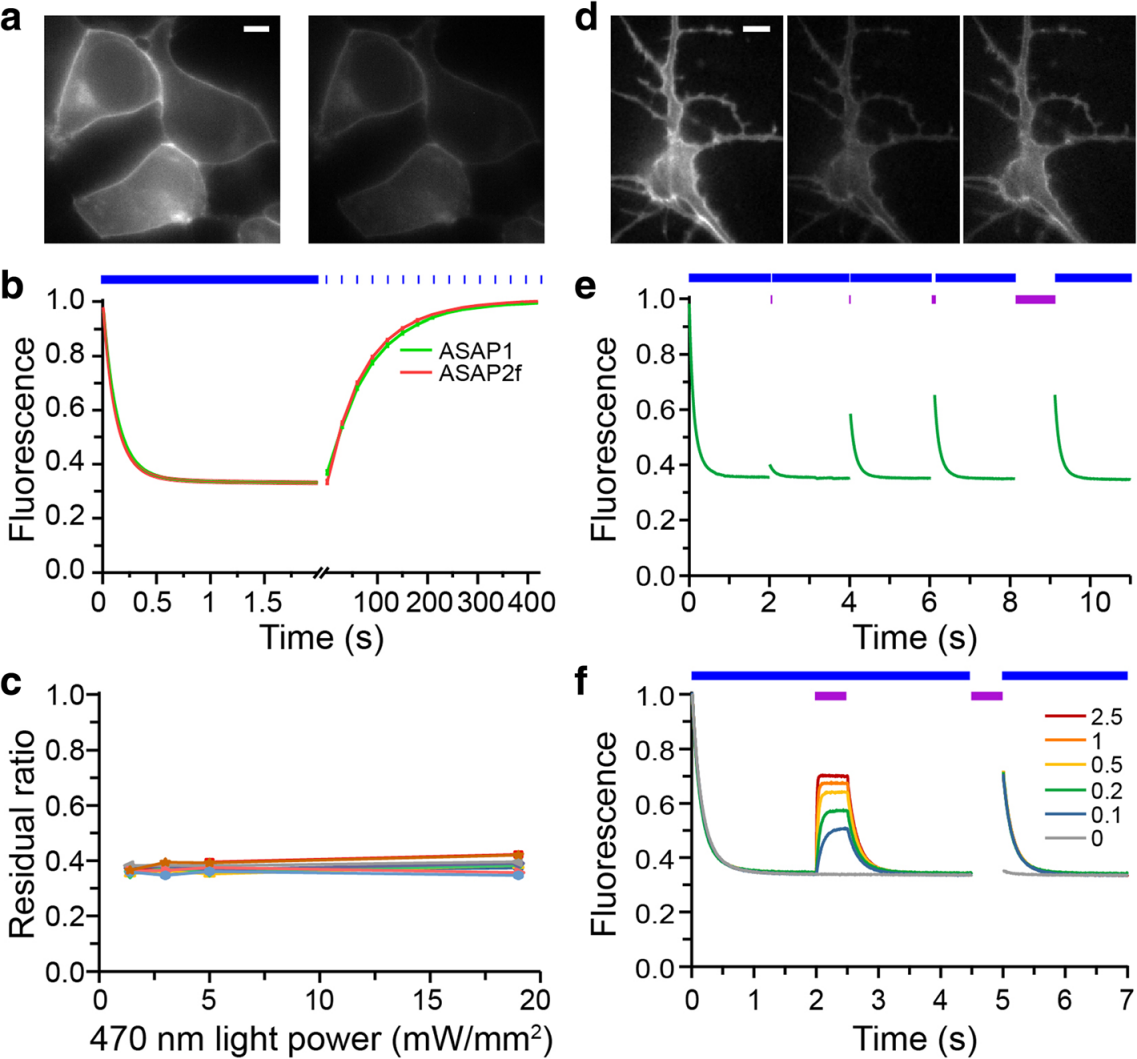
Photoswitching of ASAPs and light-induced restoration. **a** An example of fluorescence loss of ASAP1 in the transfected HEK 293 cells. *Left*, the initial fluorescence; *right*, fluorescence after 2-s 470-nm light illumination. Scale bar: 5 μm. **b** Photoswitching and spontaneous recovery kinetics of ASAP1 and ASAP2f fluorescence in transfected HEK 293 cells. Blue bars indicate duration of 470-nm light illumination. Fluorescence intensity was sampled once every 30 s after the initial 2-s continuous imaging. Error bars are SEM (*n* = 25 for ASAP1; *n* = 38 for ASAP2f). **c** The ratio of residual ASAPs fluorescence after photoswitching by 470-nm light at different power density (not significant for any pairs, t-test; *n* = 9 cells). **d** Reduction and restoration of fluorescence in a cultured hippocampal neuron transfected with ASAP2f. *Left*, the initial fluorescence; *middle*, fluorescence after 2-s 470-nm light illumination; *right*, fluorescence after 0.5-s 405-nm light illumination. **e** Photoswitching of ASAP2f fluorescence in a neuron excited by 470-nm light (blue bars) illumination was restored by interleaved 405-nm light pulses (purple bars) lasting 1, 10, 100, 1000 ms, respectively. **f** Photoswitching of ASAPs was partially rescued by assistive 405-nm light co-illumination at 0.1~ 2.5 mW/mm^2^. After 0.5-s 405-nm illumination at 0.1~ 2.5 mW/mm^2^ alone, fluorescence was partially rescued and followed by photoswitching in 470-nm illumination

To avoid errors caused by minor asynchronization of illumination and image acquisition, the initial fluorescence was calculated from the fitting curve. The fluorescence was fitted with a bi- exponential decay function:

$$ f(t)={A}_0+{A}_1\ast {e}^{-\frac{t-{t}_0}{\tau_1}}+{A}_2\ast {e}^{-\frac{t-{t}_0}{\tau_2}}, $$

where *A*_0_, *A*_1_, *A*_2_, *t*_0_, *τ*_1_, *τ*_2_ are fitting coefficients and. *f*(*t*_0_) = *A*_0_ + *A*_1_ + *A*_2_ was calculated as the initial fluorescence, and *f*(+∞) = *A*_0_ was calculated as the residual fluorescence. Relative fluorescence was normalized to the calculated initial fluorescence. For evaluating the ASAP response to APs, fluorescence was calibrated by subtracting the corresponding values of the fitting function and then divided by those values to get a corrected relative ratio (Fig. 1g). For Fig. 1b where decay time constants were evaluated, the fitting curve was simplified as a mono-exponential function by setting *A*_2_ = 0. For evaluating the recovery time constants, similar mono-exponential functions were also used (Fig. 1b and Fig. 4c).

## Results and discussion

ASAPs retain the fluorescent properties of cpGFP, which emits green fluorescence when excited by blue light. We found that ASAPs exhibited fluorescence loss with biphasic kinetics when exposed to blue excitation light centered at 470-nm (Fig. 1a), with the rapid phase lasting less than one second followed by a near-steady state with very slow photobleaching (Fig. 1b). At 5 mW/mm^2^, a light intensity typically used in voltage detection, ASAP1 and ASAP2f lost ~ 2/3 of their initial fluorescence intensities in the rapid phase (ASAP1: 67.2 ± 0.3%, *n* = 25; ASAP2f: 67.4 ± 0.5%, *n* = 38; mean ± SEM, Fig. 1b). Altering light intensity from 1.5~ 20 mW/mm^2^ did not affect the magnitude of fluorescence loss in the rapid phase (Fig. 1c). This fluorescence loss, unlike irreversible photobleaching caused by chromophore destruction, was reversible and recovered completely in the dark (Fig. 1b). The spontaneous recovery could be fitted with a mono-exponential function, with time constant of ~ 1 min (ASAP1, 58.5 ± 1.2 s, n = 25; ASAP2f, 51.8 ± 0.5 s, n = 38), much larger than the time constant of rapid dimming following blue light illumination. We observed similar effects in HEK293 cells and cultured hippocampal neurons transfected with ASAPs. It is noticeable that the photobleaching curve is different with that in the original study of ASAP2f (Fig. S2 of reference [18]). A likely explanation is the ASAP2f bleach curves in the original paper were done with exposure times 4 s apart and the fast photoswitching was over before the first image was taken, while in our current study the acquisition was synchronized with the illumination and was continuous.

The reversible fluorescence loss appeared similar to photoswitching events reported in some other GFP variants such as rsEGFP [21]. Such photoswitching may be caused by cis-trans isomerization, which enables transition between anionic (~ 470-nm-absorbing) and neutral (~ 400-nm-absorbing) states of GFP variants [22]. As these photoswitching GFP variants show recovery to the anionic state upon 400-nm illumination [22, 23], we tested whether this was the case with ASAPs. Indeed, brief 405-nm illumination restored some of the fluorescence loss induced by 470-nm illumination (Fig. 1d). The extent of restoration depended on the duration of 405-nm illumination, with durations up to 100-ms restoring increasingly more fluorescence, reaching a maximum effect of about half of the lost fluorescence, resulting in a level about 1/3 below the initial fluorescence (Fig. 1e). Recovered fluorescence could be switched off again by subsequent 470-nm illumination, falling to the same steady-state intensity as before (Fig. 1e). When applying 405-nm and 470-nm illumination simultaneously, the magnitude of fluorescence restoration depended on the intensity of the 405-nm light. For 5 mW/mm^2^ 470-nm light, the rescuing effect of 405-nm co-illumination saturated at ~ 2.5 mW/mm^2^ (Fig. 1f), resulting again in a level about 1/3 below the initial fluorescence (ASAP1: 37.2 ± 0.3%, *n* = 25; ASAP2f: 37.6 ± 0.5%, *n* = 23). The result that not all switched fluorescence is recoverable may be due to 405-nm illumination also causing some reversible photobleaching, or some chromophores in the trans state being deprotonated and thus not absorbing at 405 nm.

**Fig. 2:**
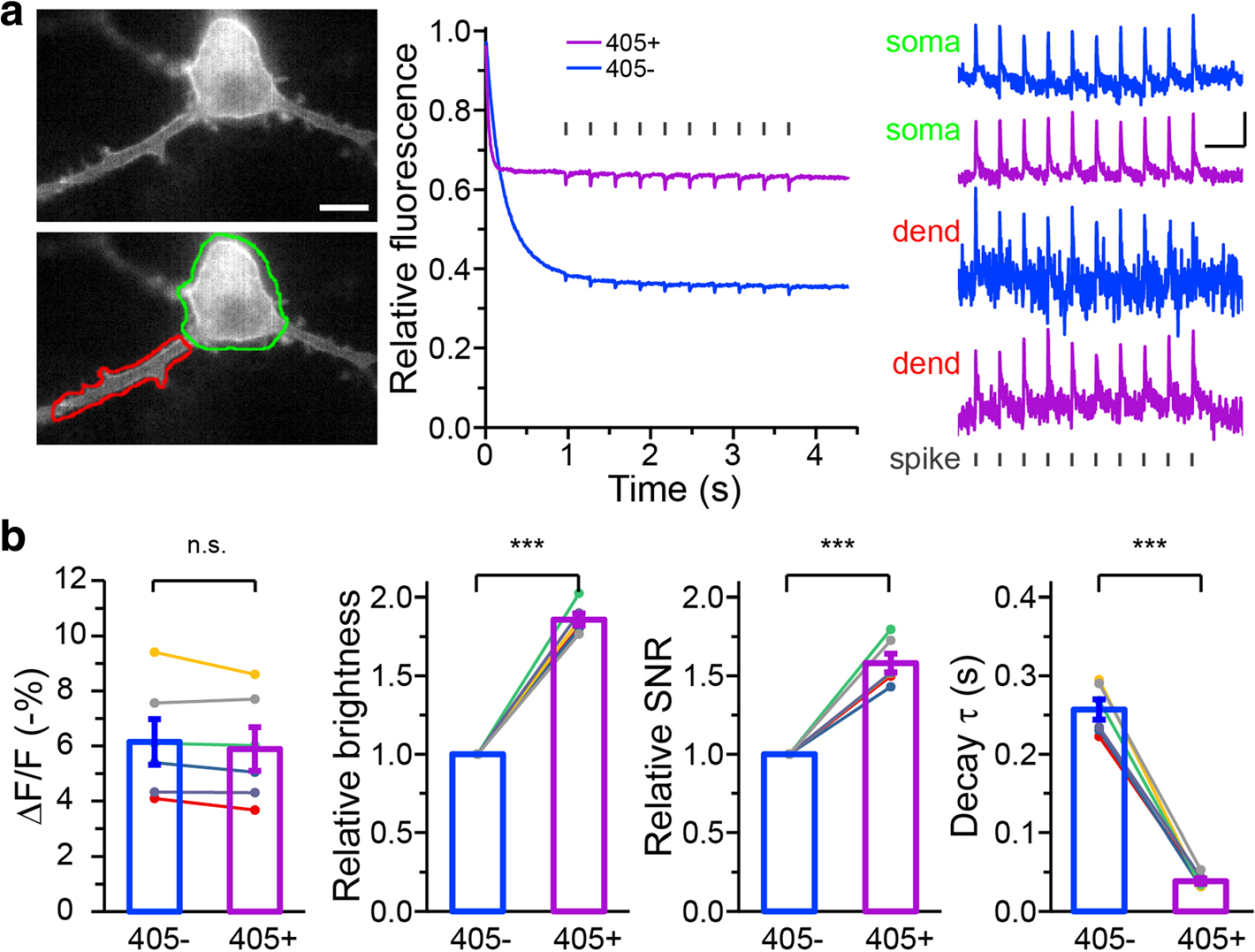
Improved photostability and performance of ASAP2f with assistive illumination. **a** (*left*) An example image showing a hippocampal neuron expressing ASAP2f driven by current pulses (2 ms, 1600pA, 5 Hz; 22 pulses) delivered via a patch pipette to induce APs. Fluorescence intensity at soma (green ROI) and dendrite (red ROI) was measured separately. Scale bar: 5 μm. (*middle*) Example traces showing soma fluorescence change in response to APs without (−) or with (+) 405-nm assistive illumination. Spike timings are indicated by short vertical bars. (*right*) Amplified view of relative fluorescence change in soma and dendrite (dend) without (blue) or with (purple) 405-nm light illumination. Scale bar: − 3%, 0.5 s. **b** (*left to right*) Summary of fluorescence changes (ΔF/F), relative brightness and relative SNR in response to single APs, and decay time constant of fluorescence in ASAP2f transfected neurons with (+) and without (−) assistive 405-nm illumination. (***) indicates *p* < 0.001, paired t-test (*n* = 6); error bars are all SEM. In all experiments, light intensity of 470-nm and 405-nm light was 5 mW/mm^2^ and 0.5 mW/mm^2^, respectively. Exposure time was 5 ms for all images

Since assistive 405-nm co-illumination significantly increased ASAPs fluorescence, we expected that 405-nm co-illumination would benefit SNR for measuring voltage transients with ASAP. To test this, we co-illuminated cultured hippocampal neurons expressing ASAP2f with 5 mW/mm^2^ 470-nm and 0.5 mW/mm^2^ 405-nm light (Fig. 2a). Assistive 405-nm light markedly improved the spike-related ASAP2f signal as represented by the relative fluorescence change ΔF/F, compared to 470-nm illumination alone. While the fluorescence response of ASAP to single action potentials (APs) remained unchanged with 405-nm co-illumination (without 405-nm light, − 6.1 ± 0.8%, *n* = 6; with 405-nm light, − 5.9 ± 0.8%, *n* = 6), the relative brightness and SNR were significantly improved (relative brightness with 405-nm light, 1.86 ± 0.04; relative SNR with 405-nm light, 1.58 ± 0.06; *n* = 6) compared to 470-nm illumination alone (Fig. 2b). Even with very weak 405-nm light illumination (e.g. 0.2 mW/mm^2^), significant improvement could be achieved, with ~ 40–50% improvements in brightness and SNR (Additional file 1: Figure S1). Improved performance allowed more reliable voltage detection in subcellular areas, such as back-propagating APs in dendrites (Fig. 2a). Additionally, we noticed a shorter decay time before the fluorescence loss reached its steady state (without 405-nm light, 0.243 ± 0.012 s; with 405-nm light, 0.036 ± 0.003 s; *n* = 6) (Fig. 2b).

**Fig. 3:**
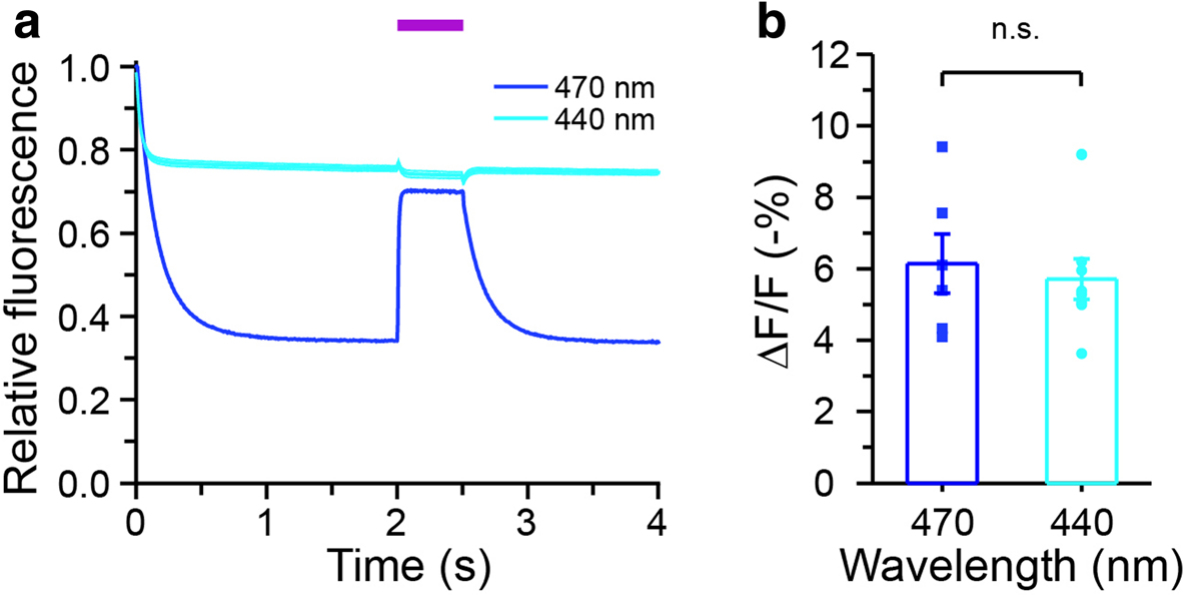
Improved photostability of ASAP2f with single 440-nm illumination. **a** Unlike 470-nm illumination, 440-nm light elicited much less photoswitching of ASAP2f in transfected neurons and was not further improved by assistive 405-nm co-illumination (indicated by a purple bar). **b** Fluorescence response of ASAP2f to single APs remained unchanged under 440-nm illumination (440-nm: - 5.7 ± 0.6%, *n* = 7; 470-nm: - 6.1 ± 0.8%, *n* = 6; n.s.: *p* > 0.05, *t*-test)

Studies of photoswitching in other GFP variants suggest that there might be two main states of the ASAPs, a 470-nm-absorbing cis anionic state and a 400-nm-absorbing trans neutral state [22]. We hypothesized that an intermediate wavelength between 400-nm and 470-nm may be able to excite both states and thus mimic the effects of dual illumination with 405-nm and 470-nm light. We indeed verified that ASAP2f was also excitable with 458-nm and 488-nm laser light, producing similar emission spectra (Additional file 1: Figure S2). We then tested the photostability of ASAP2f upon illumination by a single 440-nm LED. Remarkably, fluorescence loss of ASAP2f was much lower than that upon 470-nm illumination alone, reaching steady state with 75.6 ± 0.7% of initial fluorescence remaining (*n* = 7; Fig. 3a). The kinetics was also much faster, similar to that with 405-nm and 470-nm co-illumination. Interestingly, 405-nm co-illumination no longer improved photostability of ASAP fluorescence when imaged with 440-nm light. Compared to 470-nm, 440-nm illumination also did not alter voltage responsivity of ASAP (Fig. 3b). Thus, 440-nm illumination is a simple and convenient method for improving the photostability of ASAPs during voltage imaging.

**Fig. 4:**
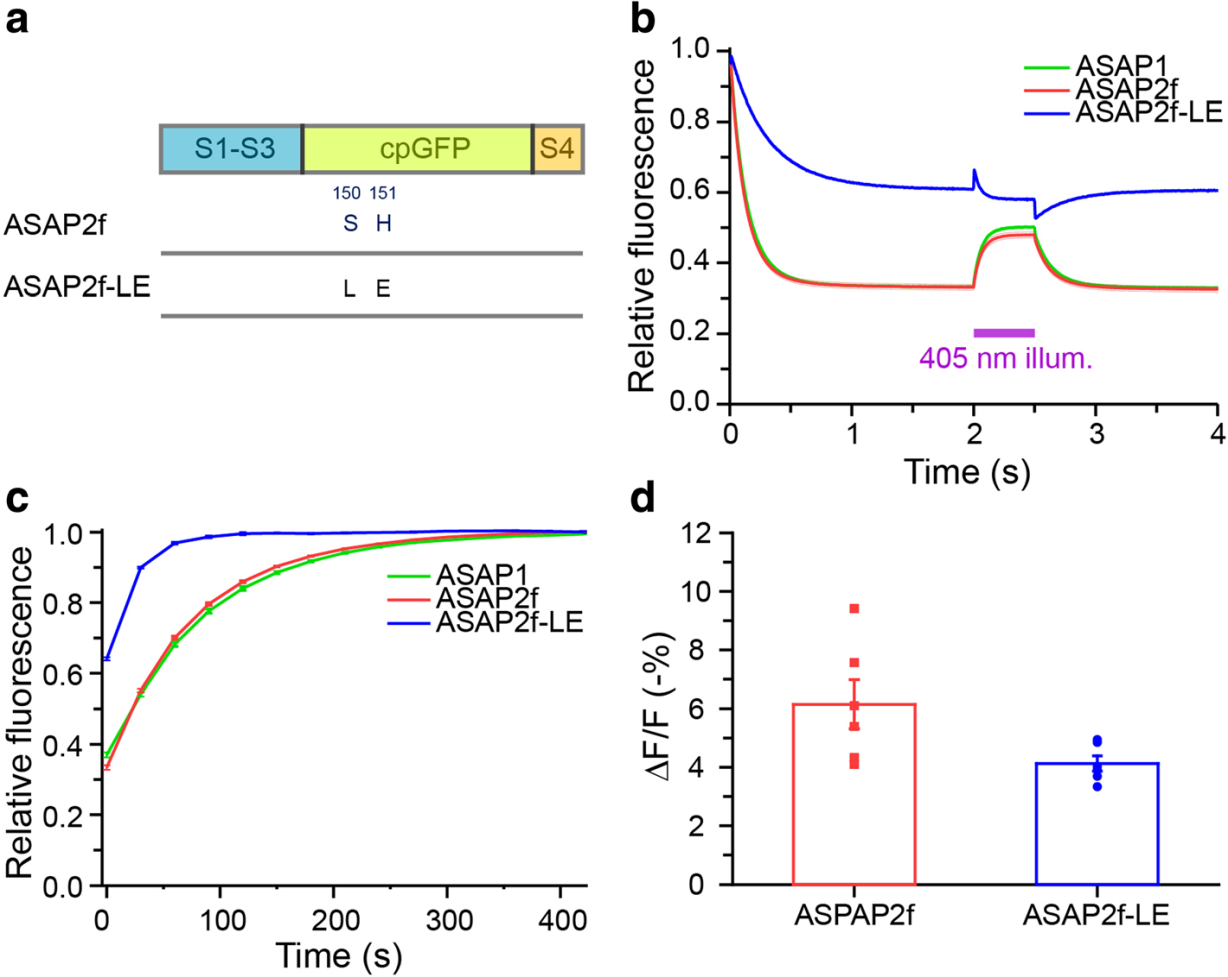
Mutations at linker sites improved ASAPs photostability. **a** Schematic diagram showing the constructs of ASAP2f and its mutant ASAP2f-LE. **b** ASAP2f-LE mutant expressed in neurons showed less photoconversion compared to original ASAP1 and ASAP2f. The fluorescence loss of ASAP2f-LE was no longer recoverable by 405-nm co-illumination. **c** Spontaneous recovery from photoswitching of the mutant in the dark. All imaging conditions were similar to that in Fig. 1b. **d** ASAP2f-LE showed lower voltage sensitivity in response to single APs. *p* = 0.04, *t*-test; *n* = 6 for ASAP2f, *n* = 6 for ASAP2f-LE

It is known that His-148 of GFP interacts with the deprotonated cis-chromophore of GFP and thus may stabilize the 470-nm-absorbing state [24, 25]. In GCaMPs that do not exhibit fast photoswitching, residues 144–148 of GFP are deleted and the function of His-148 is replaced by a water molecule held in place by an Arg residue of calmodulin [4]. In contrast, residues 144–148 are retained following the circular permutation site in the GFP of ASAPs. We hypothesized that mutating the chromophore-interacting sites could improve the photostability of ASAPs. As a proof of concept, we mutated positions Ser-147 and His-148 of GFP in ASAPs (Fig. 4a), which correspond to positions 150 and 151 in the full sequence of ASAP2f, to Leu and Glu. Glu in place of His at 151 (ASAP2f numbering) might be expected to reduce cis-trans isomerization due to its larger side-chain. Indeed, photostability of the mutant under 470-nm illumination was significantly improved, with 60% more residual fluorescence (ASAP2f-LE: 60.2 ± 0.3% of initial fluorescence; Fig. 4b). Interestingly, 405-nm co-illumination no longer rescued fluorescence loss, suggesting this mutant did not form a protonated chromophore upon 470-nm illumination. Photorecovery in the dark was also faster than ASAP1 or ASAP2f. (ASAP2f-LE: 16.1 ± 0.3 s; Fig. 4c). Although voltage responsivity was reduced (ΔF/F -4.1 ± 0.3%, *n* = 6, Fig. 4d), these findings demonstrate that engineering of ASAPs to improve their photostability is possible.

In summary, we found that ASAP-family GEVIs, including ASAP1 and ASAP2f are photoswitchable, exhibiting rapid decay to a lower-intensity steady state upon continuous 470-nm illumination. We also noticed that ASAP2s ([13]), and newer mutants show biphasic photoswitching of similar extents (data not shown). A full characterization will be performed in future studies. We demonstrated that 405-nm co-illumination with 470-nm illumination, or a single excitation wavelength of 440 nm, reduces photoswitching and greatly improves the performance of ASAPs while preserving their voltage responsivity. This insight yields a simple and effective approach to improve brightness and thus SNR of ASAPs in voltage imaging. We think that similar mechanisms would work in longer-term experiments although this must be test in future studies. One likely explanation for the photoswitching effect is the transition between the two states that previously characterized for GFP-like proteins: a 470-nm-absorbing cis anionic state and a 405-nm-absorbing trans neutral state. In addition, we cannot rule out the possibility of a non-fluorescent anionic state that is induced by 470-nm light. The presence of this state would explain the inability of 405-nm light to fully restore ASAP fluorescence. It may also explain why the Glu-151 mutant of ASAP2f still shows some loss of fluorescence upon 470-nm light, but without the ability to be restored by 405-nm light. Our findings may be instructive for future engineering of ASAP probes with improved performance and perhaps new properties. For instance, given the precedence of irreversibly photoactivatable GFP-based probes such as PA-GCaMP [26], it may be possible to create irreversibly photoactivatible ASAP variants, which may be beneficial in applications require high contrast.

## Additional file

Additional file 1: Supporting Figures. **Figure S1.** Weak 405-nm light illumination improved ASAPs performance on AP detection. **Figure S2.** Emission spectrum of ASAP2f excited by 458-nm and 488-nm illumination. (DOCX 192 kb)

## Abbreviations

cpGFP: Circularly permuted GFP
DIV: Day(s) in vitro
eFRET: Electrochromic Förster resonance energy transfer
GEVI: Genetically encoded voltage indicator
GFP: Green fluorescent protein
SNR: Signal-to-noise ratio
VSD: Voltage sensing domain
