# Supplementary material for: Excitation wavelength optimization improves photostability of ASAP-family GEVIs
Source: Mol Brain. 2018 Jun 4;11:32. doi: 10.1186/s13041-018-0374-7 (PMC5987426; doi:10.1186/s13041-018-0374-7)
Supplement: Supplementary file 1 — Supporting Figures. Figure S1. Weak 405-nm light illumination improved ASAPs performance on AP detection. Figure S2. Emission spectrum of ASAP2f excited by 458-nm and 488-nm illumination. (DOCX 192 kb) [file 13041_2018_374_MOESM1_ESM.docx]

**Supporting Figures**

| 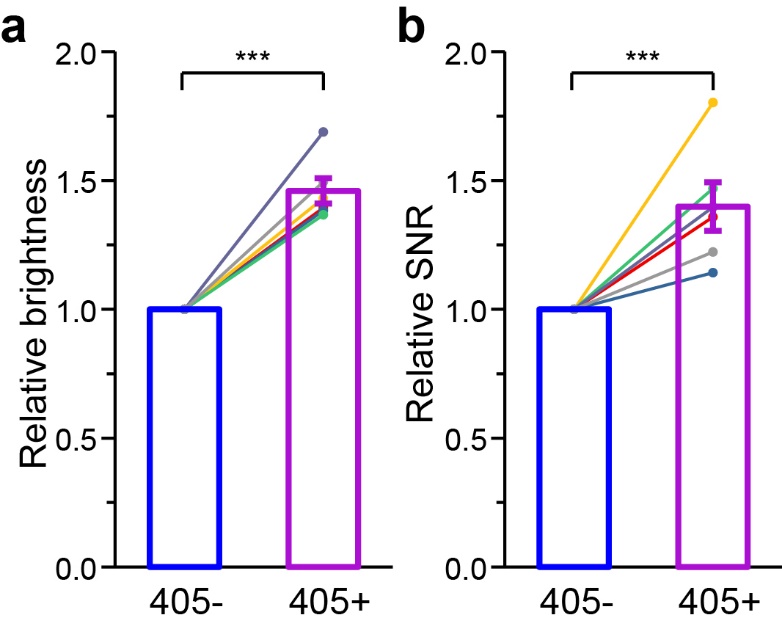 |
| --- |
| **Figure S1. Weak 405-nm light illumination improved ASAPs performance on AP detection.**  (a-b) Similar to Figure 2b, but with 0.2 mW/mm^2^ 405-nm light illumination. Both brightness and SNR were enhanced in the existence of 405-nm light. Relative brightness of 405+: 1.46 ± 0.05; relative SNR of 405+: 1.40 ± 0.09 (mean ± SEM). In both conditions, power intensity of 470-nm illumination was 5 mW/mm^2^. |

| 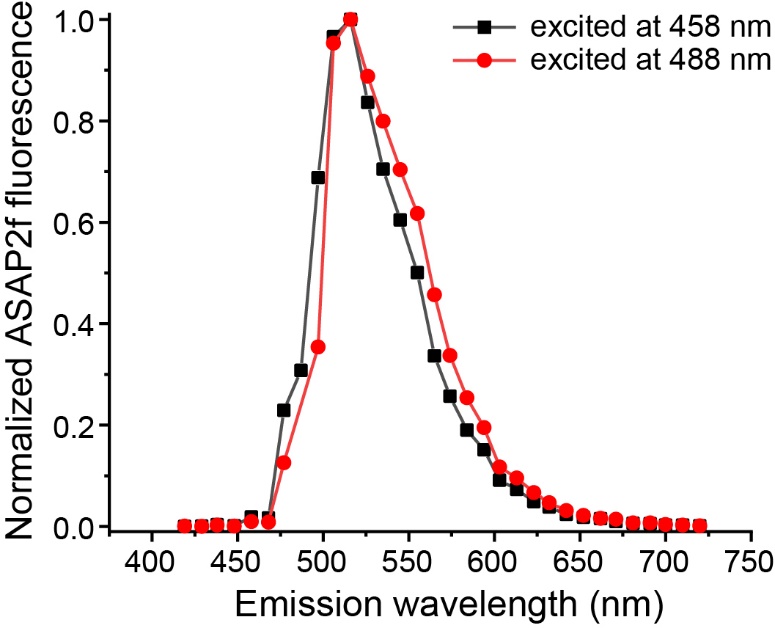 |
| --- |
| **Figure S2. Emission spectrum of ASAP2f excited by 458-nm and 488-nm illumination.** |
